# Adolescent Friendly Health Clinics (AFHCS) in India and their compliance with government benchmarks: A scoping review

**DOI:** 10.12688/f1000research.131112.2

**Published:** 2023-07-06

**Authors:** Deepika Bahl, Shalini Bassi, Subhanwita Manna, Monika Arora

**Affiliations:** 1 Health Promotion Division, Public Health Foundation of India, Gurgaon, Haryana, 122002, India

**Keywords:** Adolescent Friendly Health Clinic, AFHC, Adolescents, Youth, Rashtriya Kishor Swasthya Karyakram, RKSK, India, Review

## Abstract

**Background: **Adolescent Friendly Health Clinics (AFHCs) are one of the critical pillars of India’s Adolescent Health Programme-Rashtriya Kishor Swasthya Karyakram that seeks to enable all adolescents to realize their full potential by making informed decisions concerning their health and by accessing the services. Thus, a review was conceptualised to assess the compliance of AFHCs with the benchmark proposed by the Government under Rashtriya Kishor Swasthya Karyakram. **Methods: **Three databases (PubMed, Scopus and Google Scholar) were searched for articles published between 2014 and December 2022. A snowball search strategy was also used to retrieve all published articles. Based on the search strategy eight studies were included. **Results: **AFHCs are not fully compliant with all the benchmarks proposed by the government of India. Evidence from the primary studies showed that the benchmarks need attention as privacy was lacking (six out of seven studies), unavailability of Information Education and Communication material (four out of five), signages (two out of four), referrals (one out of two), and judgemental attitude of health care providers (one out of 3). **Conclusions: **There is a pressing need to focus on the fulfilment of these gaps to make the clinics adolescent-friendly. This might increase the utilisation of available services in AFHCs by adolescents and will improve their health. The improved health will catalyse achieving the Sustainable Development Goals indicators that are associated with nutrition, reproductive health, sexual and intimate partner violence, child marriage, education, and employment.

## Introduction

Adolescence (10-19 years) is a unique phase of life characterised by rapid changes in physical, biological, cognitive, social, and emotional development. ^1^ Investing intensively in adolescent health and development is not only crucial to improve their survival and well-being but also for India’s development and achievement of Sustainable Development Goals (SDGs) related to health, nutrition, education, gender equality, and food security. ^1^ Investment in adolescent health and wellbeing yields triple benefits, as it benefits adolescents into their future adult life and the next generation. ^2^ Health services and policies have largely ignored adolescence but, with the global HIV epidemic among adolescents, policymakers recognised the need to focus on adolescents’ sexual and reproductive health (SRH) ^3^ needs. Simultaneously, numerous other adolescent health issues were spotlighted by the Lancet series on adolescent health in 2007, ^4^ 2012, ^5^ 2019 ^6^ and 2022. ^7^ The latest 2022 Lancet series highlights the global efforts to protect every child from before conception to adulthood. ^7^

In alignment with the international focus on adolescent health, there are various adolescent health programmes being implemented like the Reproductive and Child Health (RCH) program (I and II) ^8^ and Reproductive, Maternal, Newborn, and Child Health + Adolescents (RMNCHA+A) launched in 2013. ^9^ With these sustained efforts, adolescent health improved which is evident by comparing rounds of the National Family Health Surveys (NFHS) for the reduction in adolescent childbearing, contraceptive knowledge, use of contraceptive methods, mean age at first marriage, etc. ^10^^,^^11^ While other health problems persisted such as tobacco use among adolescents, injuries and violence, mental health issues, weight gain, undernutrition, anaemia etc. ^12^ For addressing these concerns, the Ministry of Health and Family Welfare (MOHFW), Government of India (GOI) in 2014 launched a flagship programme ‘Rashtriya Kishor Swasthya Karyakram’ (RKSK). The program aimed to reach out to 253 million adolescents - male and female, rural and urban, married and unmarried, in and out-of-school adolescents with a special focus on marginalised and underserved groups. The program under its ambit has expanded the scope from Sexual Reproductive Health (SRH) to nutrition, injuries, and violence (including gender-based violence), non-communicable diseases (NCDs), mental health and substance misuse, as a one-stop-shop. The strategies include community-based, facility-based interventions, and school-based interventions. ^13^ The facility-based pillar of the programme focuses on providing adolescents with clinical and counselling services through Adolescent Friendly Health Clinics **(**AFHCs). ^14^

AFHCs are one of the critical pillars of the RKSK that seeks to enable all adolescents to realize their full potential by making informed decisions concerning their health, by accessing the services and support to implement their decisions. ^15^ Services in these clinics are being delivered through trained service providers- Medical officers (MO), Auxiliary Nurse Midwives (ANM), and Counsellors. These clinics have been integrated into medical colleges, district and sub-district hospitals, community health centres (CHCs), and primary health centres (PHCs) ^14^ to bring services ‘closer to home’ for adolescents. At a different level of the health system, manpower and timings of services at the AFHCs vary. As per GOI, there are 7969 AFHCs as of 31st Dec 2019 in India; with the highest number of AFHC in Karnataka (2563) and lowest in Chandigarh (4) at various levels of health system catering to diversified health and counselling needs of adolescents. ^16^ Since these clinics have been operational for several years; assessing compliance or alignment with the standards/indicators is critical for evaluation and considering further up-gradation if needed. With this background, a review was conceptualized to assess the compliance of the AFHCs with a benchmark proposed by MoHFW-GoI under RKSK focused on infrastructure, distance and convenient working hours, awareness about the clinic and range of services, competency of health service providers, privacy and confidentiality, awareness of the community members and referral linkage.

## Methods

### Design

To map the compliance of current adolescent healthcare facilities based on the RKSK benchmark set by GoI, ^17^ we adopted a scoping review design ^18^ by using Arksey and O’Malley’s five stage scoping review framework. ^19^

*Stage 1: Identify the research question*

Identifying the research question is the first step in carrying out a scoping review, according to Arksey & O’Malley. ^19^ Therefore, our scoping review addressed the following research question in an effort to synthesise the available knowledge in this field:

What is known about the compliance to the RKSK benchmark set by the GOI in various regions of India based on the available literature?

*Stage 2: identifying relevant studies*

Our search strategy was established after conducting preliminary research to understand search terms and keywords. We looked up the terms “AFHC” AND “Adolescent Friendly Health Clinics,” “RKSK,” “compliance,” and “India” in PubMed, Scopus, and Google Scholar ( Table 1).

**Table 1.** Search strategy.

Name of database	Search strategy	No of article
PubMed	(((“Adolescent Friendly Health Centres”) OR (“Adolescent Friendly Health Clinics”)) OR (RKSK)) OR (“Adolescent clinics”)	0
	(((compliance) OR (benchmark)) OR (“Government guideline”)) OR (“Government regulations”) OR (“Government standard”)	
	((*1) AND (*2)) AND (India)	
Scopus	(((“Adolescent Friendly Health Centres”) OR (“Adolescent Friendly Health Clinics”)) OR (RKSK)) OR (“Adolescent clinics”)	14
	(((compliance) OR (benchmark)) OR (“Government guideline”)) OR (“Government regulations”) OR (“Government standard”)	
	((?1) AND (?2)) AND (India)	
Google Scholar	“Adolescent Friendly Health Clinics” AND “benchmark”	10
Total		24

*Stage 3: Selection of literature: Inclusion criteria*

The systematic inclusion of the papers was done in accordance with the PRISMA (Preferred Reporting Items for Systematic Reviews and Meta-Analyses) recommendations. ^20^ Our search approach identified twenty-four papers, which were then imported into Mendeley desktop 1.19.5. Ten duplicate items were deleted, leaving Fourteen articles to be screened. We screened titles, abstracts, and references using the criteria described below. The last search was carried out by two researchers from July 2022 to December 2022. Benchmark of AFHCs as per GOI’s guidelines was identified as the outcome of the studies.

*3.1. Types and scope of studies*

In this review, studies using both quantitative and qualitative approaches were included from every research setting and region in India. We looked for papers that had been published in India between 2014 and December 2022 and had any information on the standard for AFHCs according to the Indian government. The studies conducted before 2014 were excluded, as that was the year the RKSK was launched. A priori, no language or publishing status restrictions were established.

*3.2. Key concepts*

Key concepts pertaining to the AFHC benchmarks established by the GOI under RKSK were covered in this review.

*3.3. Participants*

This review focused on various level health facilities which provide AFHC services to adolescents aged 10-19 years.

Following this, seven full-text papers were evaluated, and four papers were further omitted due to incorrect outcomes and full-text non-availability. Our review did not impose any geographic limitations in order to gather as many data as feasible and take diversity into account. We only considered articles that had undergone peer review. Papers without an English translation were not included in our analysis. However, a snowball search strategy was used to retrieve all maximum possible articles.

*3.4. Stage 4: Data extraction*

Data related to the benchmarks was extracted on the excel sheet and before retrieving the data, the excel sheet was piloted by the researchers of the team. Information extraction included seven benchmarks set by MoHFW-GOI including Infrastructure, clean, bright and colourful, accessibility (distance and convenient working hours), awareness about the clinic and range of services it provides (Information, Education and Communication, Proper Sinages), non-judgmental and competent health service providers, privacy and confidentiality, awareness of the services to the community members, their need and referral from the periphery/community to the higher facilities and speciality clinics. ^17^ Two reviewers (SB and DB) looked over the full texts and abstracts of all the publications as well as their citations to determine each article's eligibility. Two authors independently evaluate the articles and extracted the data for this review based on the set benchmarks of GOI. Disagreements were resolved by discussion with the third author.

*3.5. Stage 5: Collating, Summarizing and Reporting the Results*

To summarise our data, we used the author and year of publication, AFHC benchmarks, and clinic awareness. This study presents findings related to infrastructure, community awareness of the services provided, referral from the periphery/community and further referral linkages with higher facilities and specialty clinics, privacy and confidentiality, nonjudgmental and competent health service providers, and accessibility as AFHC benchmark parameters. However, clinic awareness also included the provision of Information, Education and Communication (IEC) and signage. A review synthesis was carried out, taking into account the differences between the various studies in terms of study aims, design, and conclusions.

## Results

### Selection of studies

**Figure 1.**
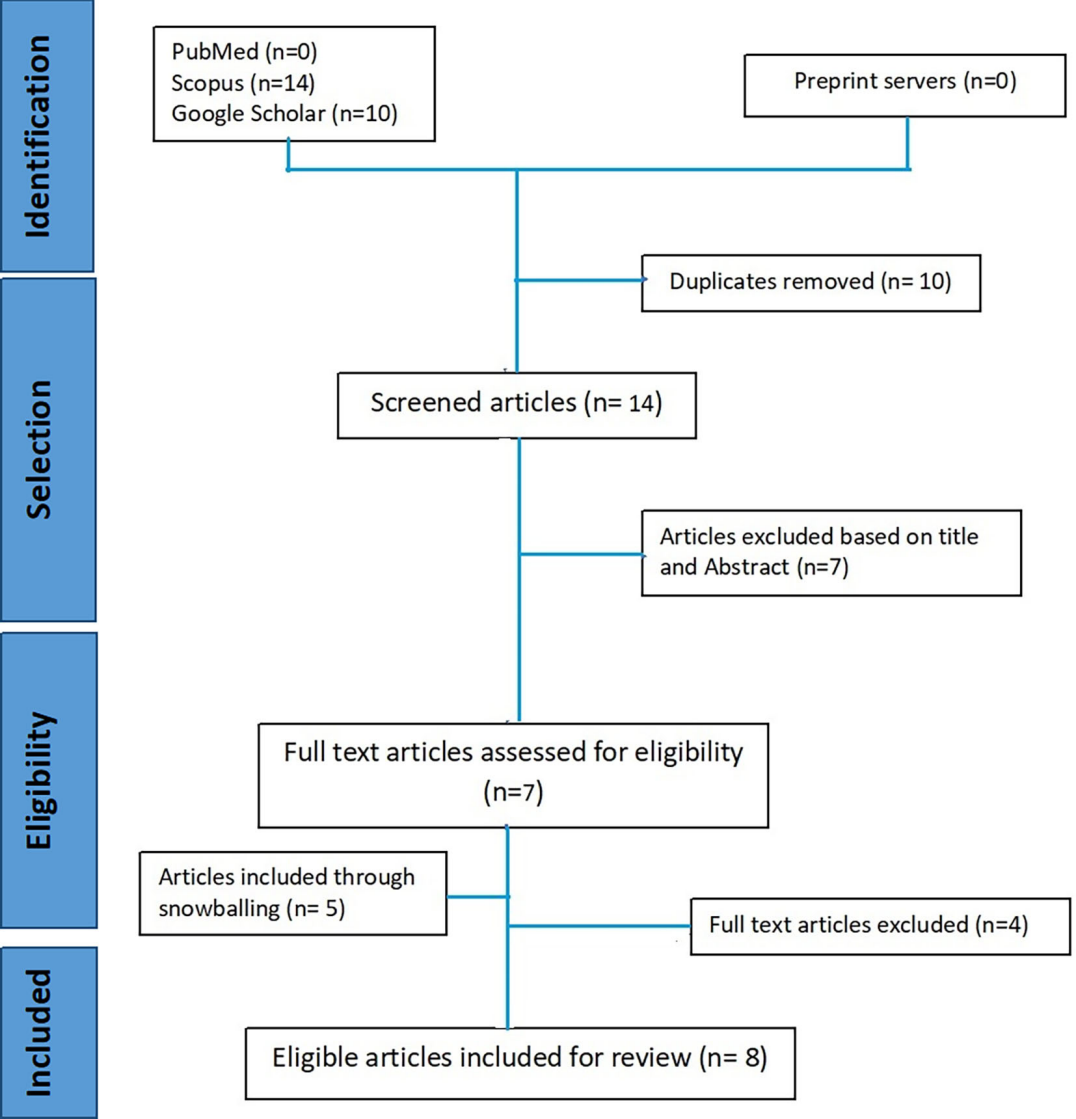
PRISMA flow diagram of included studies in review.

We found 24 papers from PubMed(n=0), Scopus(n=14), and Google scholar(n=10) in the initial database search. After removing the duplicates, 14 articles were screened, seven articles were excluded based on title and abstract screening. Only seven articles were eligible for full-text screening. While five articles were included during full-text screening by checking the references. Thus, a total of 12 articles were reviewed and finally, only eight articles were included in this review ( Figure 1).

### Characteristics of studies

Eight studies from the year 2014 to 2022 were identified and included in the review. Of eight studies, six were cross-sectional, 1 cohort and 1 qualitative was included for review. The included studies had been conducted in different geographical locations of the country like Kashmir, ^21^ Ladakh, ^21^ Leh, ^22^ Uttar Pradesh, ^23^ Ahemdabad, ^24^ Jharkhand, ^25^^,^^26^ Maharastra, ^25^ Rajasthan, ^15^^,^^25^ Madhya Pradesh, ^27^ Delhi, ^26^ Haryana, ^26^ Punjab, ^26^ and Uttrakhand. ^26^ They covered AFHC ^15^ at various levels of establishment, for example, at CHC, ^25^^,^^27^ district hospital ^21^ and sub-district hospital. ^21^^,^^25^ These studies included, either ‘exit interviews’ of patients ^23^^–^^25^^,^^27^ availing services at AFHCs or interviews of health care providers/counsellors/staff ^22^ dealing with adolescents in AFHC and ‘Mystery clients’, ^25^^,^^26^ to gather information about AFHC.

**Compliance of AFHCs with benchmarks enlisted under the RKSK**

*Privacy and confidentiality*

Out of the eight studies, seven studies evaluated the privacy and confidentiality aspect of AFHCs. Of the available seven studies, six studies reported that privacy and confidentiality in AFHCs were not maintained ( Table 2) and below is the evidence from the independent studies to support this statement.

**Table 2.** Compliance of AFHCs with MOHFW’s benchmarks.

S.No	Author and Year	Benchmark of AFHCs						Clinic awareness	
		Infrastructure (clean, bright and colourful)	Community members are aware of the services provided	Referal from the periphery/community and further referral linkages with the higher facilities and specialty clinics	Privacy and confidentiality	Non-judgmental and competent health service providers	Accessibility (distance and convenient working hours)	Availability of IEC	Availability of Signage
1	Santhya *et al.*, 2014 ^25^	Yes	Not evaluated	Yes	No	Yes	Yes	Not evaluated	Not evaluated
2	Kumar *et al.*, 2015 ^23^	Not evaluated	Not evaluated	Not evaluated	No	Not evaluated	Yes	No	Not evaluated
3	Dixit *et al.*, 2017 ^24^	Yes	Not evaluated	Not evaluated	No	Yes	Yes	No	No
4	Bhat *et al.*, 2019 ^21^	Not evaluated	Not evaluated	Not evaluated	Yes	Not evaluated	Yes	Yes	No
5	Wadhwa *et al.*, 2018 ^26^	Not evaluated	Not evaluated	Not evaluated	No	Not evaluated	Not evaluated	No	Not evaluated
6	Dolma, 2018 ^22^	Not evaluated	Not evaluated	Not evaluated	Not evaluated	Not evaluated	Not evaluated	Not evaluated	yes
7	Bali *et al.*, 2020 ^27^	No	Not evaluated	Not evaluated	No	Not evaluated	Yes	Not evaluated	Yes
8	Dayal *et al.*, 2022 ^15^	Yes	Not evaluated	No	No	No	Not evaluated	No	Not evaluated
Summary	Benchmark not maintained	1 out of 4	Not evaluated	1 out of 2	6 out of 7	1 out of 3	0 out of 5	4 out of 5	2 out of 4

AFHC - Adolescent Friendly Health Clinic; IEC - Information, Education and Communication; MOHFW - Ministry of Health and Family Welfare.

Qualitative assessment emphasised seven of the fifteen clients from Jharkhand, Maharashtra and Rajasthan felt that their conversation with the MO or other staff could have been overheard by others. In addition, four of the nine clients who underwent a physical examination felt that others could have seen them while being examined by the MO. ^25^ Similarly, interaction with mystery clients of Rajasthan reported disturbance from the staff and other patients visiting the clinic during their counselling sessions. In four out of 24 visits, these clients reported that someone was present (besides the practitioner/counsellor) during the sessions. In as frequent as half of the visits (12/24), the clients reported people coming in between the counselling sessions. This was mainly due to the location of the clinic which led to disturbance and visibility by others. ^15^

Quantitative analysis in the identified studies also showed that confidentiality and privacy were breached. The majority of adolescents (90.91%) did not receive privacy during the consultation and curtains were available only during counselling of 42.42% of adolescent girls ^24^ during their visits to AFHCs of Ahmedabad. During the clinic's observation, privacy and confidentiality were provided in only 63.6% of the facilities. ^24^ Similarly, in RKSK facilities of Madhya Pradesh, curtains were not there (33%) and conversation could be heard from outside (40%). ^27^ A six-state (Delhi, Haryana, Himachal Pradesh, Jharkhand, Punjab, Uttrakhand) evaluation of AFHCs showed that only 35% of them maintained privacy; with the least being in AFHCs of Himachal Pradesh (0%). ^26^ Adolescents (69%) from Ahmedabad believed that there should be an opportunity for separate private discussions with doctors at AFHCs. ^28^

On the contrary, there was only one study from Kashmir and Ladakh that showed privacy was maintained. Results showed that 75% of AFHCs operational under RKSK had a dedicated space and the consultation room ensured the privacy of the clients. ^21^

*Infrastructure (clean, bright and colourful)*

Four out of eight studies did not evaluate the AFHC infrastructure like cleanliness and brightness. The remaining four studies evaluated this benchmark and three studies reported that AFHC infrastructure was maintained well and in only one study the benchmark was not maintained, below is the evidence.

As per the qualitative results from a study conducted in three states, all the adolescents found the facility to be clean. ^25^ Similar results were reported by all the adolescents (100%) accessing the AFHC in Ahmedabad. ^24^ In another study 17 out of the 24 i.e., 70% of adolescents reported the clinic to be ‘overall clean from inside and outside highlighting cleanliness in these clinics. ^15^ On other hand, a study conducted in 10 districts of Madhya Pradesh showed that only 60% of the clinics were clean. ^27^

*Non-judgmental and competent health service providers*

Another benchmark of the AFHC is to have non-judgemental and competent healthcare providers. Out of eight studies, only three studies have highlighted the finding around this benchmark. Out of three studies, one study did not meet the benchmark.

One-third of the adolescents in a study reported some counsellors’ behavior was “judgemental and biased” as the counselor had a judgemental attitude towards unmarried adolescent girls engaging in premarital sexual activity. ^15^

Although, in one study almost all adolescent clients, regardless of their sex, or state of residence, reported that the healthcare provider had greeted them cordially when they entered the consultation room (21 of the 24 visits). ^25^ Similarly, in another study adolescents of Ahmedabad reported that 93.9% of health care providers were welcoming during their visits. ^24^

*Accessibility (distance and convenient working hours)*

The accessibility of these clinics is defined by the distance and the convenient working hours. Out of eight studies, three studies did not evaluate the accessibility of AFHCs. The remaining five studies evaluated this benchmark, and all (n=5) showed that the clinics were accessible to adolescents, below is the supportive evidence.

According to the result of the study conducted in Ahmedabad, 87.8% of adolescents reported that time and days are convenient to seek services ^24^ and similarly 100% of adolescents reported that time was convenient. ^23^ Similarly, when evaluating the AFHC of Kashmir ^21^ and Madhya Pradesh it showed that they were functional on all days. ^27^ Concerning the distance, AFHC was 5-10 kilometers away from the villages and also the respondents reported that these facilities were easily accessible. ^25^

*Awareness about the clinic and range of services (IEC, Proper Signages)*

Another important variable is awareness of the clinic assessed through signboards and IEC material displayed in the AFHC. Out of eight studies, three studies did not evaluate this benchmark. Of the five studies, four studies showed that the IEC material benchmark was not fulfilled.

Evidence from the studies showed that reading material was available but was readable by only 56.5% of adolescents and less than half of adolescents (43.4%) reported that the material was interesting. ^29^ In results derived through observations in another study, no IEC material was displayed in the AFHC. ^24^ AFHC evaluation from six states of India showed that only 44% of clinics displayed material. ^26^ Similarly, the adolescents reported that only limited IEC material on SRH was displayed in the waiting area of all the AFHCs. ^15^

On the contrary, only one evaluation from Kashmir and Ladakh regions showed a majority (83.3%) of the facilities had IEC material available. ^21^

Regarding the availability of signages, only four studies evaluated the availability of signboards, and the findings gave a mixed picture (two studies met the benchmarks and the other two did not). In one evaluation, it showed that signboards were only seen in one-fourth of AFHC ^24^ and another evaluation showed that 67% of AFHC from 10 districts of Madhya Pradesh had sign boards. ^27^ On the contrary, evaluation of AFHCs from Ahmedabad showed signboards were available in 100% of the AFHCs ^21^ and similar findings were seen when AFHCs from Jammu and Kashmir were observed. ^22^

*Referrals and community members awareness*

Community members’ awareness of the service provided has not been evaluated in the included studies. Another benchmark is the referral from the periphery/community to the higher facility. Out of eight studies, six studies have not evaluated the referral mechanism. The remaining two evaluated, out of which in one study mystery clients reported that they had been referred to a higher facility for SRH issues. ^25^ Similarly in another study, all counsellors reported referring cases of substance abuse, alcohol addiction, STIs, menstrual pain, unwanted pregnancy, and nocturnal emissions to more qualified healthcare providers but the adolescents reported only a few of them were referred to doctors by the counsellors for nocturnal emissions, STIs and teenage pregnancy. ^15^

## Discussion

This review sets out that existing AFHCs are not fully compliant with all the benchmarks proposed by MOHFW, GOI under the National Adolescent Health Programme. The benchmarks that need consideration include privacy in these clinics (six out of seven studies), availability of IEC material (four out of five studies), availability of signages (two out of four studies), referrals (one out of two studies), and non-judgemental attitude of health care providers (one out of three studies). It is important to fill these gaps in the existing AFHCs, especially during the current context of COVID-19 in India due to which the adolescents have become more vulnerable. As per the brief published in 2021, adolescents are vulnerable to a spectrum of issues due to restriction measures that have been put in place such as the closure of schools, restrictions on movement, physical distancing, mask-wearing, and restricted social gatherings. ^30^ The risk of child marriage in India has heightened as a result of the pandemic’s economic fallout, cybercrimes including cyberbullying due to excessive internet use, ^31^ increased mental health issues due to information floated on social media, significant proportions of which were unverified or false. ^32^ The screen time has also increased and physical activity has dwindled among adolescents. ^30^ To overcome health and development issues, AFHCs can be a one-stop clinic as they are intended to be the first point of contact between an adolescent beneficiary and the formal healthcare system. ^33^

Thus, AFHCs strengthening is crucial for developing the trust of adolescents in availing the services at AFHCs as they are located in their vicinity. Multiple efforts such as a mechanism involving centre, state, block, and village level stakeholders for the constant AFHC evaluations and addressing the gaps identified in each evaluation are needed. In India, a study was previously conducted to evaluate the quality of Adolescent Reproductive Sexual Health services; it assessed if these services met the National Standards of care and utilized periodic program improvement recommendations through the WHO - quality assessment (QA) tools. Periodic interventions resulted in improving the average facility score from 27% to 83% and overall standards score from 28% to 81% at baseline and end line survey, respectively. ^33^ This method can be used across all Indian states to assess the functionality of AFHC at all facility levels and timely and tailor-made intervention can be given to fill the existing lacunae. United Nations agencies’ help can be sought in restructuring the AFHCs and this model has been used in other countries as well, like Tanzania. A study conducted in Tanzania described the challenges identified by the Ministry of Health and Social Welfare (MOHSW) and were then addressed with the support of WHO, UNFPA, and other partners in the country. ^34^ Similar methodologies can be adopted in India to strengthen the health system.

There is also a need for regular booster training of healthcare workers on the importance of maintaining these benchmarks proposed by GOI. This training should give special emphasis on the maintenance of privacy, confidentiality, the importance of displaying the IEC material, signages, the non-judgemental attitude of health care providers, and the importance of referrals. Maintenance of privacy is a crucial aspect of adolescent healthcare as they encourage beneficiaries to seek care and disclose sensitive information that allows the service providers to deliver appropriate clinical services. ^35^ Multiple studies have found associations between confidentiality practices and receipt of recommended services. ^36^^–^^38^ Additionally, privacy is particularly important for the issues like SRH, and substance misuse, given the possible sensitivity and stigma associated with them. ^39^ IEC is a critical step toward a social behavior change communication approach that motivates people to adopt and sustain healthy behaviors ^40^ in a short time. ^41^ Training is effective and this was evident from the findings of the Tarunya project that provided cascading ARSH training to government staff at secondary care facilities and strengthened outreach activities to enhance community engagement. After 5 years of implementation, the project evaluation showed intervention efforts contributed to improvement in the quality and initial use of ARSH services. The performance of health facilities was appreciated by clients. ^42^ Another aspect that needs attention is the non-judgemental attitude of the health providers. According to the World Health Organization, ^41^ if an adolescent feels unwelcome, it is unlikely that they would utilize the services. Adolescents are an age group that needs to be treated with respect and dignity. The aforementioned characteristics determine the friendliness and acceptability of services provided to adolescents, and hence their propensity to utilise the available services. ^43^ Capacity building of service providers in providing counselling services tailored to the age, life stage and health problem. Thus, there is a dire need for the booster training of the service providers at AFHCs to strengthen their skills to provide services in non-judgemental and sensitive manner. Another concern was the signboards and the IEC materials in the AFHCs. A marked signboard indicating if the facility has an AFHC and the timing as an important indicator as per the RKSK operational guidelines. With the presence of these signages, it is easy for adolescents to locate. ^44^ The presence of this will help in adolescent knowledge enhancement. IEC materials can be Charts/Posters/Leaflets/Wall paintings, etc. on six thematic areas of RKSK and other adolescent health issues. ^45^ The presence of IEC in all the clinics will be a valuable addition, as they are a means of imparting knowledge, disseminating health information, and positively modifying health attitudes and behaviours ^46^ in a short period.

For the training of health care providers existing systems can be used. For instance, the Karnataka state involves mentoring network for the capacity building of health care providers for quality care. Across India, 900 health professionals were connected to NIMHANS ECHO. ^47^ Multi-point video conferencing was used to conduct virtual sessions with healthcare providers to improve their self-efficacy, further enhancing their interest and optimism in helping persons with addiction. This included case-based learning and didactic lecture by experts, also seeking clarification regarding standard management. Through this, 500 doctors from the district and PHC have been trained. ^47^ These existing establishments can be used as an opportunity for organizing booster trainings.

Though the robust methodology used in this review but there are some limitations including the less number of studies that were available across the different geographies of India which limited the generalizability of the study. Additionally, not all the studies included in this review have measured the benchmarks, making it challenging to replicate these findings and results must be used with caution.

## Conclusion

AFHC is one of the key pillars of the facility-based approach under RKSK and further strengthening the AFHC might increase the utilization of the available services by adolescents and will improve their health. The improved health of our young population will also help in achieving Sustainable Development Goals indicators that are associated with nutrition, reproductive health, sexual and intimate partner violence, child marriage, education, and employment.

## Authors contribution

DB and SB conceptualized the idea. DB and SB conducted the screening and data extraction. DB and SM design the methodology. DB, SB and SM drafted the manuscript and MA critically reviewed the manuscript. All the authors approved the final version of the manuscript.

## Data Availability

Figshare: Adolescent Friendly Health Clinics (AFHCS) in India and their compliance with benchmarks: A scoping review. Doi: https://doi.org/10.6084/m9.figshare.22132040.v3. ^48^ This project contains the following underlying data in the extended dataset:
- Data file Figure 1: PRISMA flowchart - Data file Appendix 1: PRISMA_ScR checklist Data are available under the terms of the Creative Commons Attribution 4.0 International license (CC-BY 4.0).
