# Supplementary material for: Adolescent Friendly Health Clinics (AFHCS) in India and their compliance with government benchmarks: A scoping review
Source: F1000Res. 2023 Jul 6;12:517. Originally published 2023 May 18. [Version 2] doi: 10.12688/f1000research.131112.2 (PMC10442587; doi:10.12688/f1000research.131112.2)
Supplement: Peer review history

# Peer review: Reviewer response for version 2

**DOI:** 10.5256/f1000research.151607.r190313

**Published:** 2023-08-21

**Authors:** Shanthi Ramasubramaniam, Roshan John

**Affiliations:** 1 University of West of England, Bristol, UK; 2 School of Health and Social Sciences, University of West of England, Bristol, UK

A well written article as this is the need of the hour for adolescent health and AFHCS in India. The review addresses a clear focus on AFHC's compliance with bench marking. Its appreciable that the Arksey and O’Malley’s five stage scoping review framework is used for this writeup. 

 However few things like why quality of studies framework like CASP was not used could have been highlighted. This could have clarified regarding the rigor of studies included in the review. Limited studies are documented and hence generalisation of results is not possible.

Are the rationale for, and objectives of, the Systematic Review clearly stated?

Yes

Is the statistical analysis and its interpretation appropriate?

I cannot comment. A qualified statistician is required.

Are sufficient details of the methods and analysis provided to allow replication by others?

Yes

Are the conclusions drawn adequately supported by the results presented in the review?

Yes

Reviewer Expertise:

My PhD topic is closely related to the article. Health Promoting behaviour Among Adolescents Attending Adolescent friendly services was my area of research. This was an action research, where I started adolescent friendly services in a private school and this was welcomed by adolescents, parents and teachers. This was the first study of its kind in 2011.

We confirm that we have read this submission and believe that we have an appropriate level of expertise to confirm that it is of an acceptable scientific standard.

# Peer review: Reviewer response for version 1

**DOI:** 10.5256/f1000research.143922.r174956

**Published:** 2023-06-08

**Authors:** Palak Gupta

**Affiliations:** 1 Utah State University, Logan, UT, USA

It is a well-written document and is of great interest in public health in India. Some of my comments are:

1. Some of the abbreviations are not explained by the author, and it will be helpful if they can be expanded upon. Example: GoI, IEC etc. IEC is expanded somewhere in the middle of the study; it will be better to explain when it is used for the first time in the manuscript.
2. The authors utilize benchmarks proposed by the Government under Rashtriya Kishor Swasthya Karyakram. It will be helpful to know what the benchmarks are, how those benchmarks are measured (which indicators are used). Also, if there is a report or website which have those benchmarks listed, adding that will be helpful.
3. This review has a limitation on the number of manuscripts available, and not all the studies have measured the benchmarks, which makes it hard to be replicated and results must be used with caution.

Are the rationale for, and objectives of, the Systematic Review clearly stated?

Yes

Is the statistical analysis and its interpretation appropriate?

I cannot comment. A qualified statistician is required.

Are sufficient details of the methods and analysis provided to allow replication by others?

Yes

Are the conclusions drawn adequately supported by the results presented in the review?

Yes

Reviewer Expertise:

Public health, Nutrition, Hunger, food and nutrition insecurity

I confirm that I have read this submission and believe that I have an appropriate level of expertise to confirm that it is of an acceptable scientific standard.

# Response

**Published:** 2023-06-14

**Authors:** Deepika BAHL

**Affiliations:** Public Health Foundation of India, India

***Comment 1:*** *Some of the abbreviations are not explained by the author, and it will be helpful if they can be expanded upon. Example: GoI, IEC etc. IEC is expanded somewhere in the middle of the study; it will be better to explain when it is used for the first time in the manuscript.*

***Response:*** *The abbreviation of GOI added in the 10th line of the second para of Introduction. IEC expanded at the first place in method section and subsequently acronym IEC has been used.*

***Comment 2:*** *The authors utilize benchmarks proposed by the Government under Rashtriya Kishor Swasthya Karyakram. It will be helpful to know what the benchmarks are, how those benchmarks are measured (which indicators are used). Also, if there is a report or website which have those benchmarks listed, adding that will be helpful.*

***Response*** *: Citation added in the 1st line of Design segment.*

*All the details regarding benchmarks already exist in the data extraction segment of the manuscript.*

*“*Information extraction included seven benchmarks set by MoHFW-GOI including Infrastructure, clean, bright and colourful, accessibility (distance and convenient working hours), awareness about the clinic and range of services it provides (Information, Education and Communication, Proper Sinages), non-judgmental and competent health service providers, privacy and confidentiality, awareness of the services to the community members, their need and referral from the periphery/community to the higher facilities and speciality clinics”

***Comment 3:*** *This review has a limitation on the number of manuscripts available, and not all the studies have measured the benchmarks, which makes it hard to be replicated and results must be used with caution.*

***Response*** *: Added to the limitation section of the manuscript.*
